# In the digital world, all roads lead to Rome. But is Rome prepared?

**DOI:** 10.1590/2177-6709.24.6.007-008.edt

**Published:** 2019

**Authors:** Flavia Artese

**Affiliations:** 1 Universidade do Estado do Rio de Janeiro, Departamento de Odontologia Preventiva e Comunitária (Rio de Janeiro/RJ, Brazil).

The Americans William Straus, author and playwright, and Neil Howe, historian, published in 1991 the book *“Generations: The history of America’s future, 1584 to 2069”* ^1^, in which they proposed the idea that generations influence society. The Strauss-Howe generation theory describes a recurrent cycle of same-aged groups with specific behavior patterns that change every 20 years. According to this theory, an 80-year cycle is crucial, when every four generations is associated to a crisis that impacts the ongoing social order and creates a new one. The four generations of this century are classified as: (a) baby boomers, from 1943 to 1960; (b) generation X, from 1961 to 1981; (c) the millennials, or generation Y, from 1982 to 2004; and (d) generation Z from 2005 to the present.

The baby boomers are part of a post-war generation that were born in a period of increased birth rate and economical growth, allowing greater expenditure. An idealistic generation that defied the ongoing social, political and cultural establishments, with movements such as anti-consumerism and feminism. The generation X was born in the oil economical crisis, in a position of dissatisfaction and apathy due to the exhaustion of alternatives to capitalism. This generation was the first to be raised in a mass consumerism culture, generating an enormous impact in the next generation. Individuals of the generation Y, or the millennials, were born in a digital world and are used to social media and the Internet. They are considered functional consumers, used to find what they wish in a fast and easy manner and transfer this situation to other aspects of life. Generation Z was born in the current technology, they are more adaptive and cannot live without a smartphone and social media. The way of sharing information by social network creates a group of individuals more concerned with concepts such as collaborative economy, environment-friendly purchase and sustainability. Nevertheless, it is too early to establish the general behavior of generation Z.

In the dusk of this decade, I can notice that the four generation theory seems to be predictable, and in 2025 we will be arriving at the end of the births of generation Z, while presently we are watching the maturing of generation Y. The changes in social behavior are clear, especially in relation to how generation Y deals with information. Until 100 years ago information was spread by printed media, which itself was a revolution for humanity, making knowledge widely available. Then, other medias such as cinema, radio, television, and more recently, the Internet, were developed^2^. It is impossible not to notice the significant acceleration in exchange of ideas and information, and this was embraced by society as an excellent marketing tool - to the point that even scientific journals were stimulated to create their own pages in social networks, to be reached by the younger public^3^.

The *Special Article* of this edition, dealing with the ethical and legal aspects of the use of digital media, has been very well explained by Dr. Alexandre Simplício. It is pointed out that there is clear inconsistency between the recent regulations of the Brazilian Dental Council and the federal laws that rule the practice of Dentistry in Brazil, as well as the Consumer Defense Code. The text by Dr. Simplício is very enlightening and of extreme importance for users of social media as a tool for professional advertisement. However, this subject not only calls my attention to those who disclose patient images in digital media, but also to the amount of digital information that we are gathering as health professionals, and how this can be used.

A few years ago, Charles Duhigg wrote an article in the *New York Times Magazine* entitled *“How companies learn our secrets”* ^4^. In this article, he describes how Andrew Pole, an employee of a chain of stores in the USA, was able to develop a formula with which, if a client bought or had interest in 25 products, she was pregnant. With this algorithm, discount coupons for pregnancy or baby products were sent to the potential clients. Shortly thereafter, the father of a teenager went to one of these stores to complain that he had received these coupons, and returned a couple of days later to apologize, since he was unaware of a few facts that had happened in his house, and his daughter would have a baby in a few months. This story became a landmark in the way digital data gathered by companies can influence society, to the extent of deducing information absolutely private to an individual. This episode opened an ethical debate in relation to the privacy laws of the USA.

In the health area, new technologies allows us to create records, photos, radiographs and impressions, all in a digital manner. This allowed an easier communication and an extraordinary capacity of data storage. The way the world is changing, the fact of having the birth registries of a child might allow, in the future, that their parents automatically receive communications and advertisements, when this child reaches 7-8 years of age, to look for an orthodontist^2^. What is lawfully allowed to be disclosed? What are the laws that regulate this privacy area? Recently the Brazilian federal government established the General Law of Personal Data Protection, which also includes the data that health providers produce^5^. The revolution of digital communication allows us, with simple clicks, to reach and disclose an endless amount of information. In this digital world, never before the proverb *“All roads lead to Rome”* has made so much sense. What remains to be know is: is Rome prepared? I don’t believe so, and I trust in generations Y and Z to tackle this important and ethical mission.
